# Acute pancreatitis promotes the generation of two different exosome populations

**DOI:** 10.1038/s41598-019-56220-5

**Published:** 2019-12-27

**Authors:** A. Jiménez-Alesanco, M. Marcuello, M. Pastor-Jiménez, L. López-Puerto, L. Bonjoch, M. Gironella, M. Carrascal, J. Abian, E. de-Madaria, D. Closa

**Affiliations:** 1 Department of Experimental Pathology, Institut d’Investigacions Biomèdiques de Barcelona, Consejo Superior de Investigaciones Científicas (IIBB-CSIC), Institut d’Investigacions Biomèdiques August Pi i Sunyer (IDIBAPS), Barcelona, Spain; 2 Gastrointestinal & Pancreatic Oncology Group, Centro de Investigación Biomédica en Red de Enfermedades Hepáticas y Digestivas (CIBERehd)-IDIBAPS-Hospital Clínic de Barcelona, Barcelona, Spain; 3 Proteomics Facility, Institut d’Investigacions Biomèdiques de Barcelona (IIBB), Consejo Superior de Investigaciones Científicas/Universitat Autònoma de Barcelona (CSIC/UAB), Institut d’Investigacions Biomèdiques August Pi i Sunyer (IDIBAPS), Barcelona, Spain; 4 Pancreatic Unit, Department of Gastroenterology, Hospital General Universitario de Alicante, Instituto de Investigación Sanitaria y Biomédica de Alicante (ISABIAL - Fundación FISABIO), Alicante, Spain

**Keywords:** Extracellular signalling molecules, Acute pancreatitis

## Abstract

Exosomes are small extracellular vesicles that act as intercellular messengers. Previous studies revealed that, during acute pancreatitis, circulating exosomes could reach the alveolar compartment and activate macrophages. However, proteomic analysis suggested that the most likely origin of these exosomes could be the liver instead of the pancreas. The present study aimed to characterize the exosomes released by pancreas to pancreatitis-associated ascitic fluid (PAAF) as well as those circulating in plasma in an experimental model of taurocholate-induced acute pancreatitis in rats. We provide evidence that during acute pancreatitis two different populations of exosomes are generated with relevant differences in cell distribution, protein and microRNA content as well as different implications in their physiological effects. During pancreatitis plasma exosomes, but not PAAF exosomes, are enriched in the inflammatory miR-155 and show low levels of miR-21 and miR-122. Mass spectrometry-based proteomic analysis showed that PAAF exosomes contains 10–30 fold higher loading of histones and ribosomal proteins compared to plasma exosomes. Finally, plasma exosomes have higher pro-inflammatory activity on macrophages than PAAF exosomes. These results confirm the generation of two different populations of exosomes during acute pancreatitis. Deep understanding of their specific functions will be necessary to use them as therapeutic targets at different stages of the disease.

## Introduction

Acute pancreatitis is a frequent illness, being the 3rd leading cause of hospitalization due to gastrointestinal disease^1^. It is an inflammatory process of the pancreas that, in the severe forms, is related to systemic inflammatory response and organ failure^2^. Currently there is no specific treatment available for pancreatitis beyond supportive care^3^. This fact points to an incomplete understanding of the pathological mechanisms involved in the disease. From the first description of acute pancreatitis in 1895, it has been evaluated a number of mediators potentially involved in the progression of the disease from the local pancreatic damage to systemic inflammation. Along these years, hydrolytic enzymes, hormones, oxygen free radicals, cytokines, bioactive lipids and virtually any known molecule that can be suspected to have a role in the process, have been analyzed^4–7^. Unfortunately, this knowledge has not turned into the development of useful pharmacological treatments for the management of the disease.

Some of the lost pieces of this puzzle involve those mediators that do not circulate in a soluble form but packaged in microvesicles, including extracellular vesicles and exosomes. Exosomes are small extracellular vesicles that range between 30–200 nm in diameter that act as intercellular messengers by transferring signaling molecules, including proteins, small RNAs and lipids, to target cells. A number of studies have provided evidence of their implication in the pathogenesis of several diseases, being cancer and inflammation the fields in which exosome research has more expanded the last years^8^. In particular, the role of exosomes in the progression of acute pancreatitis from the local damage to the systemic inflammation has been described^9,10^.

In previous studies, using an experimental model of taurocholate-induced acute pancreatitis in rats, we reported the generation and release to the bloodstream of a particular population of exosomes with the ability to reach and activate alveolar macrophages^9^. Interestingly, the proteomic analysis of exosomes purified from plasma of rats with acute pancreatitis did not showed the presence of pancreatic proteins. By contrast, it revealed a significant amount of proteins of supposedly hepatic origin, including apolipoproteins, mannose-binding protein or hemoglobin subunits. These observations suggest that, during acute pancreatitis, liver could be the source of exosomes that activate the inflammatory response in the lung. On the other hand, although we identified in the ascitic fluid the presence of exosomes allegedly released by the pancreas, it still remains to determine its composition and its potential role in the systemic effects of acute pancreatitis.

Here we characterized the two different populations of exosomes generated in an experimental model of acute pancreatitis in rats. The first one, purified from ascitic fluid, is generated by the pancreas while the second population of exosomes, collected from plasma, appeared to be released by the liver. We observed that, in addition to remarkable differences in protein and micro-RNA (mi-R) content, these exosomes showed significant differences in body distribution as well as in their physiopathological roles.

## Results

### Acute pancreatitis promotes inflammation in pancreas and lung

**Figure 1:**
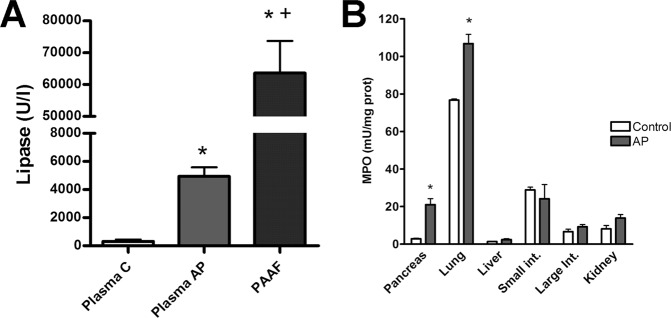
(**A**) Lipase in plasma from control (C) or acute pancreatitis (AP) animals and ascitic fluid (PAAF) and (**B**) myeloperoxidase (MPO) activity in different tissues 3 h after induction of acute pancreatitis. *p < 0.05 vs control;^+^p < 0.05 vs AP.

Pancreatitis results in a significant increase in lipase activity in plasma and also induces the peritoneal accumulation of PAAF containing high amounts of lipase (Fig. 1A). In PAAF, lipase activity was tenfold higher than levels observed in plasma. Inflammation, measured as increases in MPO activity, was observed only in pancreas and lung while other analysed tissues (Liver, kidney, small intestine and large intestine) showed no significant changes (Fig. 1B).

### Exosomes in plasma and PAAF during acute pancreatitis

**Figure 2:**
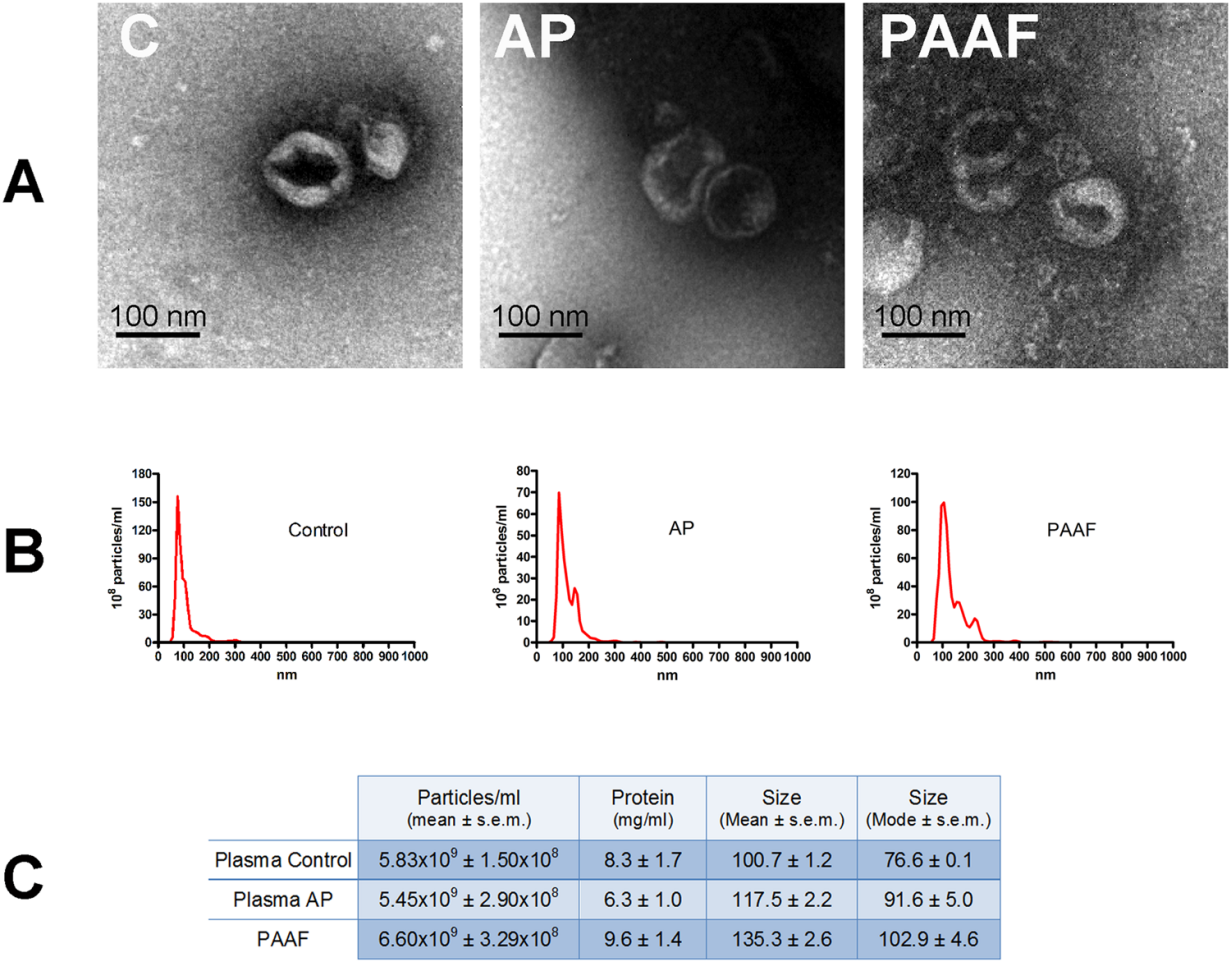
(**A**) Representative electron microscopy images of exosomes purified from plasma control (C), plasma from pancreatitis (AP) and Pancreatitis associated ascitic fluid (PAAF). (**B**) Nanovesicle tracking assay of extracellular vesicles. (**C**) Total number, protein concentration and size of particles obtained in plasma control, pancreatitis and PAAF.

We have taken advantage of the fact that, in the experimental model of taurocholate-induced acute pancreatitis in rats, mediators released by the pancreas can be collected from PAAF. This allows us to analyse separately the exosomes coming from the pancreas and those that are circulating in the blood which are generated by other organs such as the liver. Extracellular vesicles isolated from both plasma and PAAF were morphologically characterized by transmission electron microscopy (TEM). The majority of vesicles had the classical exosome appearance of spherical bubbles or “cups” with the characteristic central depression, an artifact attributed to TEM sample preparation^11^. The observed size was around 100 nm, as expected in the exosomes (Fig. 2A). This was confirmed by nanosight tracking analysis (Fig. 2B). The average of mode size of exosomes was 76.6 ± 0.1 nm from plasma control; 91.6 ± 5.0 nm for plasma from pancreatitis and 102.9 ± 4.6 nm for PAAF, which agree with the size expected for exosomes. No significant differences were detected in total particle concentration and protein content (Fig. 2C).

### Exosomes circulating in plasma are distributed to distant organs

**Figure 3:**
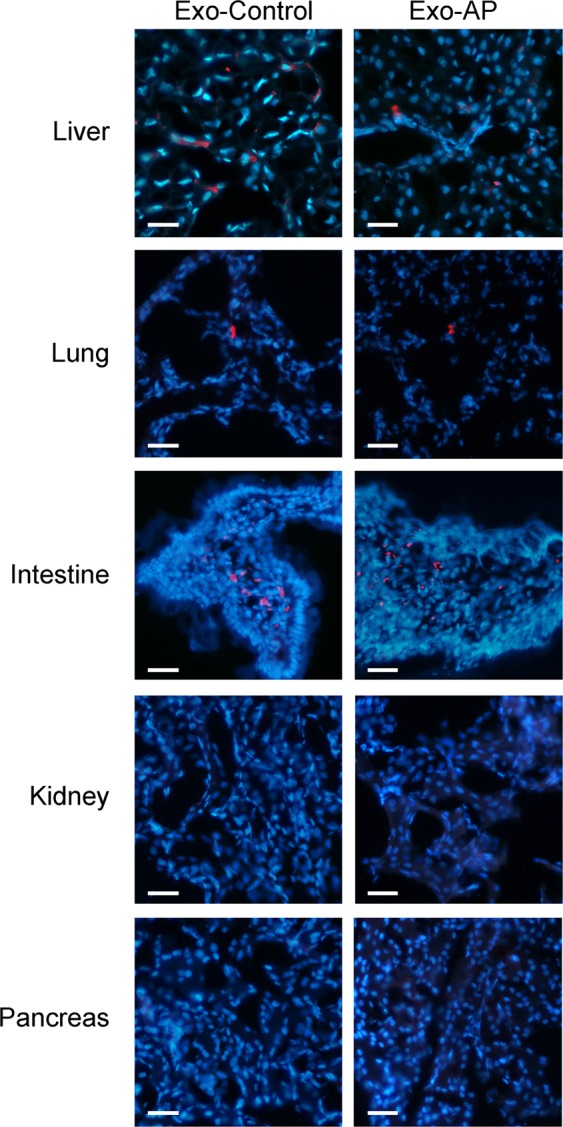
Exosomes obtained from Control and AP plasma samples were stained with PKH-26 dye (red) and perfused through the inferior vena cava. Tissue samples were obtained 30 min after administration. Nuclei were stained with DAPI (blue) (Scale bar: 50 µm).

To study the body distribution of circulating exosomes from plasma, vesicles obtained from control or pancreatitis animals were stained with PKH26 and injected into the cava vein of control rats. Thirty minutes later the presence of red fluorescent signal was detected in liver, lung and intestine. By contrast, they were not detected neither in the kidney nor pancreas. Noteworthy, there were no differences in organ distribution between control and AP exosomes (Fig. 3).

### Hepatic capture of PAAF exosomes

**Figure 4:**
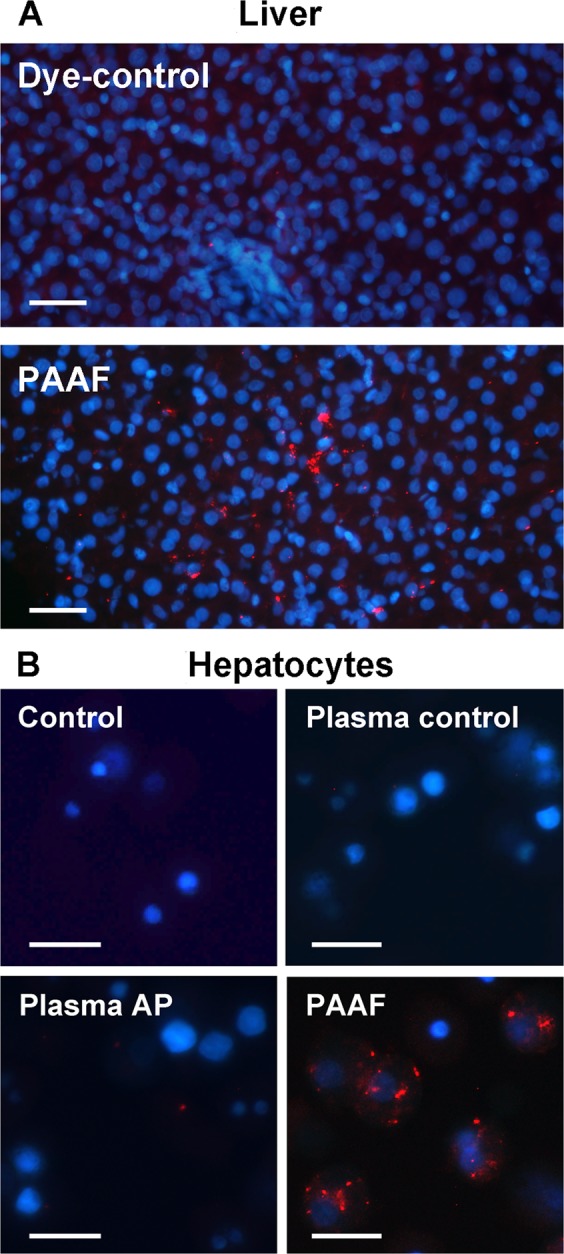
(**A**) Exosomes from PAAF were stained with PKH-26 dye (red) and perfused through the portal vein. PBS stained with PKH26 has been infused as a Dye-control group. Samples were obtained 30 min after administration. Nuclei were stained with DAPI (blue) (Scale: 50 µm). (**B**) Isolated hepatocytes were incubated *in vitro* for 30 min. with exosomes (red) from plasma control, plasma AP or PAAF. Control group was incubated with PBS stained with PKH26 (Scale: 20 µm).

As we have previously reported, PAAF exosomes stained with PKH-26 red fluorescent dye and perfused through the portal vein were retained by the liver and uptaken by hepatocytes (Fig. 4A). We also compared, in hepatocytes cultured *in vitro*, the uptake of exosomes obtained from plasma or PAAF. Results indicate that hepatocytes incorporated significantly higher amount of exosomes when they came from PAAF in comparison to the level of uptake observed when the exosomes were obtained from plasma control or plasma pancreatitis (Fig. 4B).

### PAAF-exosomes and plasma-exosomes carry different miRNAs

**Figure 5:**
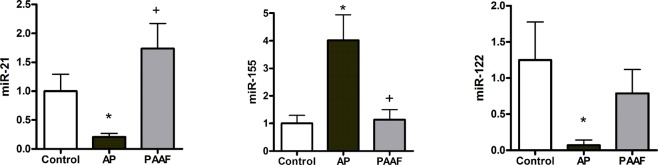
After induction of acute pancreatitis, exosomes from plasma transport higher levels of miR-155 and lower levels of miR-21 and miR-122. By contrast, no differences in those miRNAs were observed when comparing exosomes from PAAF and from plasma control. *p < 0.05 vs control; ^+^*p* < 0.05 vs AP.

Since the transfer of miRNA between cells is emerging as one of the main functions of exosomes, we evaluated the differences in the levels of three miRNAs in PAAF exosomes and plasma exosomes. The analysis revealed that after induction of acute pancreatitis, plasma exosomes contained increased amounts of the pro-inflammatory miR-155 and reduced levels of miR-122 and miR-21 in comparison to exosomes from plasma control. On the other hand, the levels of these miRNAs in PAAF exosomes were similar to that observed in plasma control (Fig. 5).

### Proteomic analysis reveals differences in exosome cargo from plasma and PAAF

To evaluate the differences in exosome protein cargo between plasma and PAAF during acute pancreatitis, a mass spectrometry-driven proteomics analysis was performed. A total of 151 proteins were identified with high confidence, including CD81, commonly found in exosomes (Supplementary Table S1). The Gene Ontology Annotation term “extracellular exosome” was found significantly overrepresented in this collection where up to 70% of the proteins were annotated under this cellular compartment (Supplementary Table S2).

The comparative analysis showed that after pancreatitis induction exosomes in plasma were enriched in a number of proteins presumably expressed by the liver (Apolipoproteins, C-reactive protein, Retinol binding protein, Alpha-2-macroglobulin…) (Table 1). Moreover, only two specific pancreatic proteins were detected (Chimotrypsinogen > 2.2 fold and amylase > 1.9 fold). On the other hand, exosomes obtained from PAAF showed a completely different protein profile. In this case, relevant enrichment (>10 fold) of several pancreatic proteins (Trypsin, Chymotrypsinogen, amylase, pancreatic elastase…) was detected but the higher enrichment was observed in histones (>30 fold) and ribosomal proteins (>20 fold) (Table 2).

**Table 1:** Differentially expressed proteins found between exosomes obtained from plasma AP respect to plasma C 3 h after induction of acute pancreatitis.

Protein ID	Protein name	Ratio	*p*-value
P01946	Hemoglobin subunit alpha-1/2	4.00	0.00E + 00
P02091	Hemoglobin subunit beta-1	2.88	1.39E − 18
P23562	Band 3 anion transport protein	2.66	1.90E − 03
P07338	Chymotrypsinogen B (EC 3.4.21.1)	2.28	2.31E − 05
P08932	T-kininogen 2	2.21	1.22E − 07
P09006	Serine protease inhibitor A3N (Serpin A3N)	2.12	2.10E − 66
P11517	Hemoglobin subunit beta-2	2.08	4.45E − 27
P04785	Protein disulfide-isomerase (EC 5.3.4.1)	2.07	2.99E − 03
P00689	Pancreatic alpha-amylase (EC 3.2.1.1)	1.95	2.66E − 04
P02764	Alpha-1-acid glycoprotein	1.66	3.31E − 17
P02761	Major urinary protein	1.64	6.62E − 05
P01015	Angiotensinogen (Serpin A8)	1.53	8.04E − 04
P48199	C-reactive protein	1.52	5.73E − 15
P01048	T-kininogen 1	1.45	3.99E − 02
P06238	Alpha-2-macroglobulin	1.43	6.20E − 03
P23680	Serum amyloid P-component	1.43	1.61E − 04
P04916	Retinol-binding protein 4	1.36	2.13E − 02
P02650	Apolipoprotein E (Apo-E)	1.33	2.17E − 11
P14630	Apolipoprotein M (Apo-M)	1.32	1.27E − 02
P02767	Transthyretin	1.24	3.65E − 11
P04937	Fibronectin	1.23	4.72E − 05
P17475	Alpha-1-antiproteinase (Alpha-1-antitrypsin)	1.21	1.65E − 64
P04276	Vitamin D-binding protein	1.20	6.85E − 12
P10959	Carboxylesterase 1C (EC 3.1.1.1)	1.19	7.14E − 09
P36953	Afamin	1.14	4.76E − 05
Q9WUW3	Complement factor I (EC 3.4.21.45)	1.13	3.99E − 02
P05545	Serine protease inhibitor A3K (Serpin A3K)	1.12	7.37E − 04
P14480	Fibrinogen beta chain	1.10	3.21E − 04
P20059	Hemopexin	1.08	1.65E − 06
P05544	Serine protease inhibitor A3L (Serpin A3L)	1.08	1.65E − 06
P31211	Corticosteroid-binding globulin (CBG)	1.08	2.68E − 02
P06399	Fibrinogen alpha chain	1.07	2.29E − 02
P02770	Serum albumin	1.05	1.28E − 10
Q03626	Murinoglobulin-1	1.03	3.96E − 03
P12346	Serotransferrin	1.02	1.45E − 02
P01026	Complement C3	0.97	1.52E − 04
P14046	Alpha-1-inhibitor 3	0.95	5.03E − 05
P01835	Ig kappa chain C region, B allele	0.92	1.65E − 02
Q99PS8	Histidine-rich glycoprotein	0.89	2.04E − 03
Q62862	MAP kinase kinase 5 (MAPKK 5) (EC 2.7.12.2)	0.84	4.30E − 03
P20762	Ig gamma-2C chain C region	0.81	1.85E − 04
Q6P6T1	Complement C1s subcomponent (EC 3.4.21.42)	0.69	4.04E − 02
Q63041	Alpha-1-macroglobulin	0.68	5.15E − 167
P02651	Apolipoprotein A-IV (Apo-AIV)	0.64	3.07E − 10
P06866	Haptoglobin	0.27	2.32E − 97

**Table 2:** Differentially expressed proteins found between exosomes obtained from PAAF respect to plasma AP 3 h after induction of acute pancreatitis.

Protein ID	Protein name	Ratio	*p*-value
A9UMV8	Histone H2A.J	30.67	0.005677
Q00715	Histone H2B	24.74	2.22E − 25
P63245	Receptor of activated protein C kinase 1	23.27	0.047568
P60868	40S ribosomal protein S20	21.79	0.001606
P62804	Histone H4	17.50	1.94E − 24
P00773	Chymotrypsin-like elastase 1 (EC 3.4.21.36)	16.94	4.65E − 11
P04797	GAPDH (EC 1.2.1.12)	15.50	2.3E − 17
P84245	Histone H3.3	13.89	0.000248
P08426	Cationic trypsin-3 (EC 3.4.21.4)	12.85	5.68E − 07
P35427	60S ribosomal protein L13a	12.83	0.001105
P02262	Histone H2A type 1	12.75	2.01E − 09
P07338	Chymotrypsinogen B (EC 3.4.21.1)	12.08	1.89E − 14
P62083	40S ribosomal protein S7 (S8)	11.37	6.49E − 06
P05197	Elongation factor 2 (EF-2)	11.32	6.65E − 09
Q5XIF6	Tubulin alpha-4A chain	11.29	0.000617
P62630	Elongation factor 1-alpha 1	11.13	1.32E − 28
P19223	Carboxypeptidase B (EC 3.4.17.2)	10.73	1.83E − 29
P00689	Pancreatic alpha-amylase (PA) (EC 3.2.1.1)	9.64	8.75E − 26
P00564	Creatine kinase M-type (EC 2.7.3.2)	8.89	2.66E − 10
P49242	40S ribosomal protein S3a	6.90	0.014226
P62250	40S ribosomal protein S16	6.57	0.007414
P09812	Glycogen phosphorylase (EC 2.4.1.1)	6.09	3.57E − 09
P06761	Endoplasmic reticulum chaperone (EC 3.6.4.10)	5.69	0.000417
P04785	Protein disulfide-isomerase (PDI) (EC 5.3.4.1)	5.45	1.67E − 06
P00774	Chymotrypsin-like elastase 2A (EC 3.4.21.71)	5.12	2.79E − 07
P02091	Hemoglobin subunit beta-1 (Beta-1-globin)	4.74	5.38E − 65
P01946	Hemoglobin subunit alpha-1/2 (Alpha-1/2-globin)	4.23	0
P15083	Polymeric immunoglobulin receptor	3.99	0.00096
P63324	40S ribosomal protein S12	3.84	0.004548
P11517	Hemoglobin subunit beta-2	3.84	6.19E − 96
P05371	Clusterin	2.83	1.35E − 16
Q64268	Heparin cofactor 2 (Serpin D1)	2.81	0.023958
P62738	Actin, aortic smooth muscle	2.78	0.039368
Q62930	Complement component C9	2.77	8.08E − 09
P06866	Haptoglobin	2.74	1.55E − 67
P23562	Band 3 anion transport protein (CD233)	2.66	6.76E − 05
P08650	Complement C5	2.42	0.000259
Q811M5	Complement component C6	2.25	0.004227
P20759	Ig gamma-1 chain C region	2.13	2.84E − 06
P04916	Retinol-binding protein 4	2.13	2.84E − 06
P55314	Complement component C8 beta chain	2.00	0.023921
Q01177	Plasminogen (EC 3.4.21.7)	1.97	1.6E − 95
Q68FP1	Gelsolin	1.93	5.27E − 05
Q7TMA5	Apolipoprotein B-100 (Apo B-100)	1.66	0.006874
P18292	Prothrombin (EC 3.4.21.5)	1.61	1.85E − 09
Q63416	Inter-alpha-trypsin inhibitor heavy chain H3	1.59	2.84E − 06
Q6P6T1	Complement C1s subcomponent (EC 3.4.21.42)	1.58	0.011707
P24090	Alpha-2-HS-glycoprotein (Fetuin-A)	1.55	7.06E − 45
P08649	Complement C4	1.53	1.43E − 17
P04937	Fibronectin (FN)	1.53	3.09E − 17
P14272	Plasma kallikrein (EC 3.4.21.34)	1.51	0.010077
P25236	Selenoprotein P (SeP)	1.50	0.00256
Q63556	Serine protease inhibitor A3M (Serpin A3M)	1.49	9.43E − 06
Q62862	MAPKK 5 (EC 2.7.12.2)	1.46	4.63E − 07
P02651	Apolipoprotein A-IV (Apo-AIV)	1.46	1.1E − 07
Q9QX79	Fetuin-B	1.45	1.23E − 20
P55159	Serum paraoxonase/arylesterase 1 (PON 1) (EC 3.1.1.2) (EC 3.1.1.81) (EC 3.1.8.1)	1.42	0.020857
Q6P734	Plasma protease C1 inhibitor (Serpin G1)	1.36	0.005552
P05545	Serine protease inhibitor A3K (Serpin A3K)	1.32	4.31E − 15
Q63514	C4b-binding protein alpha chain (C4bp)	1.31	0.003327
P14630	Apolipoprotein M (Apo-M)	1.30	0.011425
P01015	Angiotensinogen (Serpin A8)	1.30	0.018252
P20761	Ig gamma-2B chain C region	1.30	0.001105
P14480	Fibrinogen beta chain	1.29	3.04E − 21
P01835	Ig kappa chain C region, B allele	1.29	4.95E − 15
P00762	Anionic trypsin-1 (EC 3.4.21.4)	1.25	6.49E − 06
Q64240	Protein AMBP	1.24	0.005677
P05544	Serine protease inhibitor A3L (Serpin A3L)	1.24	1.33E − 35
P20760	Ig gamma-2A chain C region	1.22	0.000398
P02680	Fibrinogen gamma chain	1.19	7.24E − 09
Q9WUW3	Complement factor I (EC 3.4.21.45)	1.17	0.007751
P02650	Apolipoprotein E (Apo-E)	1.14	0.001062
P20762	Ig gamma-2C chain C region	1.14	0.016552
P17475	Alpha-1-antiproteinase (Alpha-1-antitrypsin)	1.13	1.04E − 29
Q99PS8	Histidine-rich glycoprotein	1.09	0.025281
P02767	Transthyretin (Prealbumin)	1.09	0.00852
P20059	Hemopexin	0.96	0.015278
P36953	Afamin (Alpha-albumin)	0.94	0.035576
P12346	Serotransferrin (Transferrin)	0.92	8.05E − 32
Q63041	Alpha-1-macroglobulin	0.92	8.15E − 42
P01026	Complement C3	0.90	6.76E − 57
P10959	Carboxylesterase 1C (EC 3.1.1.1)	0.89	0.000116
P06399	Fibrinogen alpha chain	0.78	3.53E − 15
P09006	Serine protease inhibitor A3N (Serpin A3N)	0.76	2.96E − 14
Q03626	Murinoglobulin-1	0.72	1E − 176
P14046	Alpha-1-inhibitor 3	0.71	3.2E − 163
P02764	Alpha-1-acid glycoprotein (Orosomucoid)	0.65	3.61E − 14
P08932	T-kininogen 2 (Major acute phase protein)	0.64	0.000121
P08934	Kininogen-1	0.64	0.000579
P23764	Glutathione peroxidase 3 (EC 1.11.1.9)	0.63	4.82E − 06
P04639	Apolipoprotein A-I (Apo-AI)	0.58	8.21E − 69
P48199	C-reactive protein	0.45	5.56E − 31
P04638	Apolipoprotein A-II (Apo-AII)	0.37	2.07E − 07
P19939	Apolipoprotein C-I (Apo-CI)	0.27	7.07E − 10

### Cellular responses to exosomes generated during acute pancreatitis

**Figure 6:**
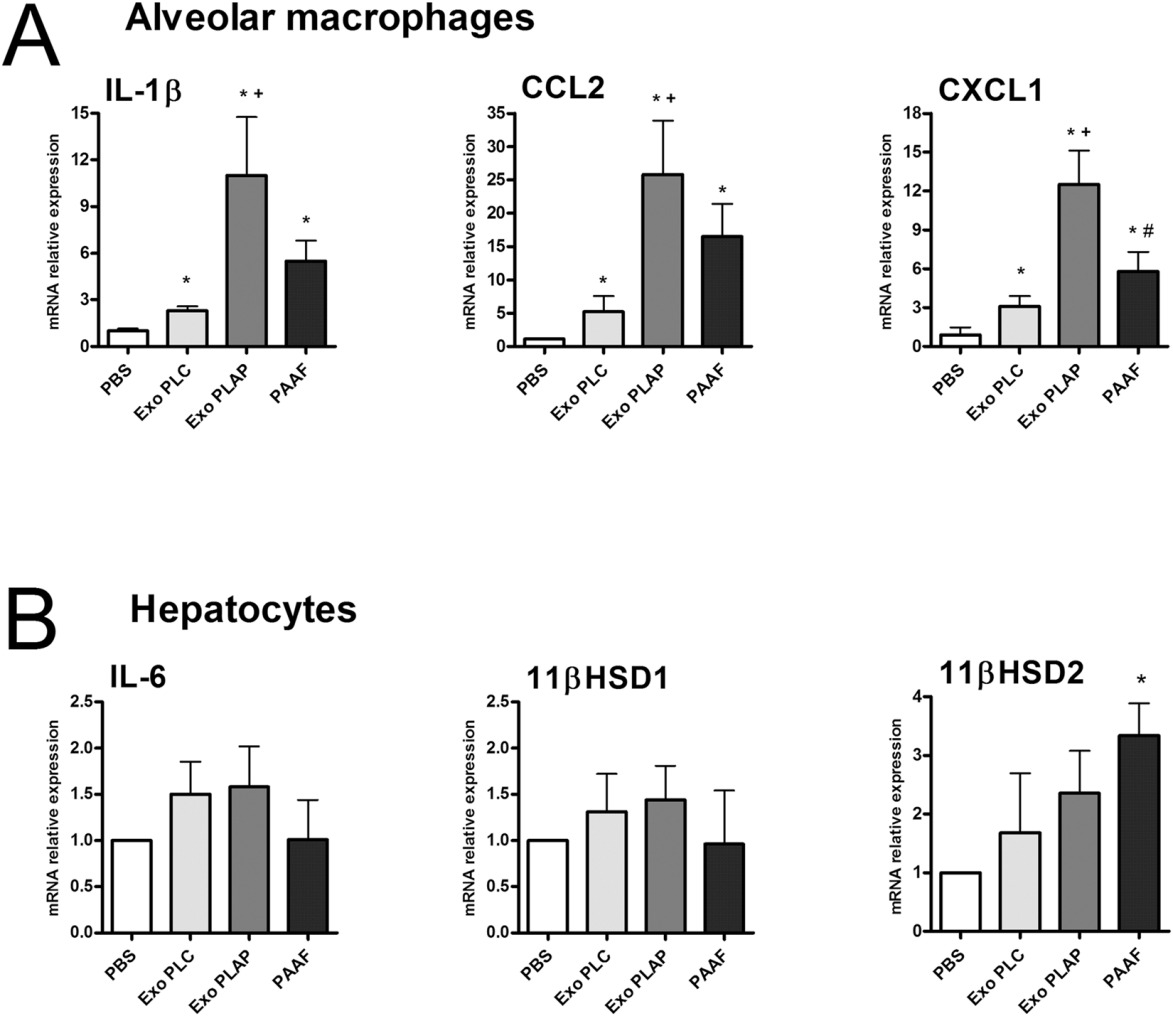
(**A**) Effect of exosomes (2 µg/ml) in macrophage activation. Even the exosomes from plasma control (exo PLC) results in a small activation of macrophages. However, exosomes from plasma AP (exo PLAP) induces higher induction of IL1β, CCL2 and CXCL1. PAAF exosomes also increases the expression of these mediators but not achieves statistical significance compared to control plasma. Control cells are treated with the equivalent volume of PBS. (**B**) In hepatocytes the only effect observed was an increase in the expression of 11βHSD2 under the treatment with PAAF exosomes. *p < 0.05 vs control; ^+^*p* < 0.05 vs Exo PLC; ^#^*p* < 0.05 vs Exo PLAP.

Since PAAF exosomes are retained in the liver while the plasma exosomes are distributed throughout the rest of the body and, moreover, they carry different content, we have analyzed whether there are also differences in the effects they have on two cell populations: alveolar macrophages and hepatocytes. In alveolar macrophages all treatments results in a small activation, however, the expression of the inflammatory cytokine IL-1β and chemokines CCL2 and CXCL1 was highly increased in response to exosomes from plasma AP. By contrast, exosomes from PAAF induces only a moderate increase that does not achieve statistical significance compared to control exosomes (Fig. 6A). On the other hand, treatment of hepatocytes with exosomes from any source did not result in relevant changes in the mRNA expression of inflammation-related genes. No changes in IL-6, LBP or 11βHSD1 expression were detected and only a moderate increase in 11βHSD2 expression was detected with PAAF exosomes (Fig. 6B).

## Discussion

Exosomes have been shown to participate in inflammatory diseases by mediating antigen presentation and carrying different proteins and miRNAs. In the case of acute pancreatitis, the role of exosomes in activation of macrophages has been previously reported *in vitro*, by evaluating the role of exosomes released by stimulated pancreatic acinar cells on macrophages^10^, as well as *in vivo*, by using the taurocholate-induced pancreatitis model in rats^9^. An unexpected conclusion of this study was the fact that the mechanisms involving the generation of these extracellular vesicles seems to be not restricted to the pancreas. Proteomic analysis of exosomes obtained from plasma in rats after induction of acute pancreatitis revealed the presence of different proteins of suspected hepatic origin and a lack of proteins with obvious pancreatic origin, pointing to the liver as a main source of the circulating exosomes.

Here we confirmed this possibility by comparing the content, distribution and physiological effects of exosomes obtained from plasma with those present in ascitic fluid of rats after induction of pancreatitis. In human patients ascites is not a frequent complication in acute pancreatitis; inflammatory exudates tend to form collections (liquid collections if necrosis is absent or necrotic collections in case of pancreatic and/or peripancreatic fat necrosis)^2^ but in the experimental models in rats the pancreatic exudate is released to the peritoneum and could be easily recovered. This PAAF contains high concentrations of pancreatic enzymes and also extracellular vesicles, including exosomes, released by the pancreatic cells. We took advantage of this to compare pancreatic exosomes, obtained from ascitic fluid, with circulating plasma exosomes, supposedly released by the liver.

In a previous work^9^ we observed that, due to the anatomical situation of the pancreas, vesicles released by this organ are collected into the portal system and circulate through the liver before reaching the systemic circulation in the vena cava. This fact is relevant for the final fate of exosomes released by the pancreas and our data confirm that they are retained in the liver (Fig. 4A). However, when we compared the affinity of the hepatocytes for the vesicles obtained from PAAF with those obtained from plasma, we observed that the absorption of pancreatic exosomes was much higher than the absorption of exosomes circulating in plasma (Fig. 4B). This difference already suggests that the two populations of vesicles present significant differences in their composition.

On the other hand, tracking experiment of exosomes obtained from plasma and injected into the cava vein revealed that they are distributed in different organs and they do not show any particular affinity for the liver (Fig. 3). In addition, there are no evident differences between the exosomes obtained from control animals than those ones obtained after the induction of pancreatitis, suggesting that there are no specific organ targets for these vesicles. Other variables, as local blood flow, could be the most relevant factors affecting the distribution of exosomes in the systemic circulation.

However, the main difference between the two populations of exosomes can be seen by comparing their cargo. Comparison between exosomes from plasma control and pancreatitis revealed that pancreatitis results in an enrichment of miR-155, and a reduction of miR-122 and miR-21 (Fig. 5). These changes were not observed in PAAF exosomes, in which the levels of these miRNAs are closely similar to those observed in the exosomes from plasma control, thus reinforcing the idea that there are two different exosome populations generated during acute pancreatitis.

Although most miRNA are multifunctional, the pro-inflammatory role of miR-155 is well known and it has been shown to promote M1 polarization of macrophages^12^. By contrary, miR-122, which is mainly produced by the liver and secreted into bloodstream, appears to play a protective and anti-inflammatory role^13,14^. Reduction of miR-122 in blood is associated with inflammation in several diseases and its deletion was shown to drive microsteatosis and inflammation^15^. The case for miR-21 is more confusing and it has been reported to play a role both in increasing and in preventing cell damage^16,17^. However, the administration of miR-21 prevented the inflammatory activation of macrophages in several models^18,19^. Therefore, our results suggest that during acute pancreatitis exosomes circulating in plasma are enriched in pro-inflammatory (miR-155) exosomes and have reduced levels of other miRNAs (miR-21 and miR-122) which could play an anti-inflammatory response, particularly in macrophages. This agree with the induction of systemic inflammatory conditions along the progression of severe forms of acute pancreatitis and could be related with the effect of these exosomes activating macrophages and promoting the release of inflammatory cytokines (Fig. 6A). Finally, it has been reported that inhibition of miR-155 improves prognosis in an experimental model of acute pancreatitis, making this miRNA a promising target for the design of therapeutical strategies for the treatment of pancreatitis^20^. Our results indicate that, in addition to the local synthesis, this miRNA could achieve the target macrophages via exosomes, providing a potential new point of control.

Even more marked are the differences in protein content (Table 1). As we have previously reported, exosomes from plasma AP are enriched in a number of hepatic proteins, including apolipoproteins, CRP or LBP when compared with exosomes from plasma control^9^. Now, the comparison between exosomes from plasma and PAAF in animals with pancreatitis revealed a completely different profile (Table 2). First, the fact that PAAF exosomes contains high levels of different pancreatic enzymes, including trypsin, amylase or chymotrypsinogen, confirms their pancreatic origin. On the other hand, the major increase detected in PAAF exosomes corresponds to some histones and ribosomal proteins, virtually absent in plasma exosomes. This fact suggests that PAAF exosomes, but not plasma exosomes, contain the nucleoprotein component TERRA (Telomeric repeat-containing RNA), a telomere-associated regulator of chromosome end protection that has been reported to be carried by exosomes from damaged cells and can elicit an inflammatory response^21^. This complex is made by a noncoding RNA transcript associated with histone proteins^22^.

We also evaluated the differences in the response of alveolar macrophages and hepatocytes to these two populations of exosomes. In alveolar macrophages the treatment with exosomes induces a response, reflected in increases in IL-1β, CCL2 and CXCL1 expression in all conditions, even with control plasma exosomes. However, the response obtained by treating the cells with exosomes from plasma AP is clearly superior, thus reflecting the pro-inflammatory activity of these vesicles. By contrast, exosomes from PAAF only results in a moderate induction, which is not statistically different to those obtained with control plasma (Fig. 6A).

On the other hand, we evaluated in hepatocytes the effect of exosomes on the expression of IL-6 and in the enzymes 11β-hydroxysteroid dehydrogenase type 1 (11βHSD1) and type 2 (11βHSD2). These enzymes were selected since they are involved in the liver metabolism of corticosterone and play a role in modulating the levels of circulating corticosterone, thus controlling the anti-inflammatory status of the organism^23,24^ and are altered during acute pancreatitis^25^. The only effect observed was an increase in the expression of 11βHSD2 induced by PAAF exosomes while no changes were detected for IL-6 or 11βHSD1 (Fig. 6B). Since 11βHSD2 oxidizes the active corticosterone (in rats) or cortisol (in humans) to the inactive cortisone, an increase in the expression of this enzyme could be related with the impaired anti-inflammatory response of the organism that occurs during pancreatitis.

Altogether, our results confirms that during acute pancreatitis, there are generated two populations of exosomes, with clear differences in their origins, tissue distribution, molecular content and physiological effects Exosomes generated by the pancreas appear to act on the liver while exosomes circulating in plasma are generated by the liver and would act as pro-inflammatory mediators activating macrophages in lung and, probably, in other systemic organs. In light of the results obtained, both populations could be interesting potential therapeutic targets for different stages of the disease. Although further studies are required to validate these results in human patients, the absence of ascitic fluid in this case will made difficult to separate the two populations of exosomes. Anyway, we can hypothesize that an early selective or non-selective removal of pro-inflammatory exosomes from plasma is a promising treatment to decrease systemic inflammation and thus, the development of organ failure, condition that is associated to a high risk of mortality^26^.

## Methods

### Animals

Male Wistar rats (250 g b.w.) (Charles River, France) were housed in a controlled environment, fed with standard laboratory pelleted formula (A04, Panlab, Spain) and tap water *ad libitum*. This study conformed to European Community for the use of experimental animals and was approved by the Institutional Committee of Animal Care and Research: CEEA-UB (Comitè Ètic d’Experimentació Animal de la Universitat de Barcelona), procedure number 10345.

### Animal model of acute pancreatitis

Animals (n = 6 each group) were anesthetized with an i.p. administration of sodium pentobarbital (50 mg/kg). After midline laparotomy, biliopancreatic duct was cannulated through the duodenum and a small bulldog clamp was used to close the hepatic duct. AP was induced by retrograde perfusion of 5% sodium taurocholate in a volume of 0.1 ml/100 g b.w. using a perfusion pump (Harvard Instruments, Edenbridge, UK)^27^. Control animals received saline solution (NaCl 0.9%). This is a very aggressive model for pancreatic tissue, but reproduces very well the systemic effects of pancreatitis. Three hours after induction, samples of blood and pancreatitis-associated ascitic fluid (PAAF) were collected and processed immediately for the isolation of exosomes. This time point was selected since at shorter induction times inflammation process in lung has not started^28^.

### Lipase activity

Measurement of plasma and PAAF lipase activity was carried out by using the turbidimetric assay kit from Randox (Antrim, U.K.), according to the supplier’s specifications.

### Myeloperoxidase activity

Neutrophilic infiltration was analyzed by measuring myeloperoxidase (MPO) activity. Samples were homogenized with 0.5% hexadecyltrimethylammonium bromide in 50 mM PBS at pH 6.0. Homogenates were disrupted for 30 s using a Labsonic sonicator (Braun Biotech, Inc., Allentown, PA), submitted to three cycles of freezing and thawing and sonicated again. Samples were incubated at 60 °C for 2 h, centrifuged at 15,000 × g for 15 min and supernatants were collected for MPO assay and protein assay. MPO activity was determined photometrically at 655 nm using H_2_O_2_ and 3,3′,5,5′-tetramethylbenzidine as substrates^29^.

### RNA isolation and qPCR

Total RNA from cells was extracted using the TRizol® reagent (Invitrogen, Carlsbad, CA). RNA was quantified by measuring the absorbance at 260 and 280 nm using a NanoDrop ND-1000 spectrophotometer (NanoDrop Technologies, USA). cDNA was synthesized from 1 µg RNA sample using the iScript cDNA synthesis kit (Bio-Rad, CA, USA).

Subsequent quantitative polymerase chain reaction (qPCR) was performed in a DNA Engine, Peltier Thermal Cycler (Bio-Rad, CA, USA) using iTaq^TM^ Universal SYBR® Green Super mix and the corresponding rat primers (Supplementary Table S3). Reactions were performed in duplicate and threshold cycle values were normalized to *GAPDH* gene expression. The specificity of the products was determined by melting curve analysis. The relative expression of target genes to *GAPDH* was calculated by the ΔC(t) formula.

### Isolation of hepatocytes and alveolar macrophages

Hepatocytes were isolated from control rats by collagenase digestion^30^, and cultured in 12 wells plates in 5% CO_2_ in air at 37 °C using Exo-Free supplemented DMEM medium (9% FBS, 100 U/ml penicillin and 100 μg/ml streptomycin)^31^.

To obtain alveolar macrophages, lungs and trachea were excised and five bronchoalveolar lavages were performed with 10 ml of NaCl 0.9%. The obtained cell suspension was centrifuged at 180 × g for 5 min and resuspended in RPMI-1640 medium containing 10% fetal calf serum, 2 mM glutamine, penicillin (100 U/ml) and streptomycin (100 µg/ml). After an attachment period of 1 h at 37 °C under a gas phase of air/CO2 (95:5), the non-adherent cells were removed.

### Exosome isolation

Exosomes were isolated as described in^32^, with some modifications. Plasma and PAAF samples (1 ml) were centrifuged at 2,000 × g and 10,000 × g for 10 and 30 min, respectively, at 4 °C. After the first centrifugation step at 2000 × g, samples were pre-treated with thrombin (5 U/ml) (Sigma, St Louis, MO) for 5 min and centrifuged at 10,000 × g for 5 min. Then, the fibrin pellet was discarded and supernatant was used for continuing the purification protocol. The 10,000 × g supernatant was recovered, resuspended in 25 ml PBS, filtered through a 0.22 µm filter and ultracentrifuged at 110,000 × g for 70 min. Pellet were resuspended in PBS and the remaining soluble proteins were eliminated by a final filtration through 30,000 mw filter. Isolated exosomes were resuspended and quantified by measuring their protein content.

### Nanoparticle tracking analysis

Quantification of concentration and size of exosomes obtained in plasma and PAAF were carried out using a NanoSight LM10 machine (NanoSight) by the ICTS “NANBIOSIS” at the ICMAB-CSIC. All the parameters of the analysis were set at the same values for all samples and three 1 min-long videos were recorded in all cases^33^. The background was measured by testing filtered PBS, which revealed no signal.

### Electron microscopy

Isolated exosomes were fixed in 4% paraformaldehyde, adsorbed in formvar-coated cooper grids and negatively stained with 2% uranyl acetate. Grids were air dried and observed in a JEOL-1010 Transmission Electron Microscope^9^.

### Exosome staining and tracking analysis

For tracking experiments, exosomes were labeled with PKH26 Red Fluorescent Cell Linker Dye (Sigma, St Louis, MO) according to the supplier’s specifications. The staining reaction was stopped with 3% BSA for 1 min, and the labeled exosomes (Exo-PKH26) were washed three times with PBS in order to remove the unbound dye, using 300 KDa Nanosep centrifugal devices (Pall Corporation). For each group, PBS stained with PKH26 was used as a control.

In a first tracking analysis, 7 µg of Exo-PKH26 obtained from Control or AP plasma samples were resuspended in 1 ml of saline solution and perfused through the inferior vena cava of control animals at a rate of 6 ml/h during 10 min as previously described^9^. After 30 min, animals were sacrificed and samples of pancreas, liver, lung, kidney and small intestine were obtained and processed for the histological analysis.

In a second experiment, Exo-PKH26 from PAAF samples were perfused to control animals through the hepatic portal vein at a rate of 4 ml/h and livers from portal-perfused animals were obtained for immunofluorescence analysis.

### Exosome microRNA analysis

Total RNA, including miRNAs, was isolated from exosomes using the Total Exosome RNA and Protein Isolation kit (Life Technologies, Foster City, CA, USA) according to the manufacturer’s protocol. In addition, we performed an optional enrichment for small RNA during the extraction in order to obtain higher quantities of miRNAs. To monitor miRNA extraction efficiency in all samples, 5 µl of synthetic *C. elegans* Cel-miR-39 (5 nM) were spiked-in in each sample before RNA extraction. We assessed exosome miRNA expression of miR-21, miR-155, miR-122 and cel-miR-39 by real-time qRT-PCR by using TaqMan microRNA reverse transcription Kit (Life technologies) according to the manufacturer’s instructions. All miRNAs except the spike-in cel-miR-39 were pre-amplified. Cycle threshold (Ct) values were calculated from automatic threshold using QuantStudioTM Real Time PCR Software. All samples showed similar extraction efficiencies according to cel-miR-39 Ct values. Due to the absence of a known endogenous miRNA in exosomes to be used for normalization, 2^−Ct^ values were used to calculate the expression of each candidate miRNA. Values were normalized to total µg of protein present in each exosome sample.

### Liquid chromatography-Mass spectrometry analysis

Protein extracts were digested with Sequencing Grade Modified Trypsin (Promega, Madison, WI) using the FASP (Filter Aided Sample Preparation) digestion protocol^34^. Each tryptic peptide mixture was labeled with tandem mass tags (TMT) (Thermo Scientific, Rockford, IL) based on the standard procedure. Six labeled peptide mixtures were combined, evaporated, and desalted using a C18 SPE cartridge (Agilent Technologies, USA). Two independent TMT-labeled assays were performed (n = 4).

Labeled samples were analyzed by LC-MSn using an LTQ-Orbitrap XL instrument equipped with a nanoESI ion source (Proxeon, Odense, Denmark) as described^35^. Database search was carried using Protein Discoverer software (ThermoFisher, San Jose, CA) against the Uniprot database restricted to rat and using the target-decoy strategy. DanteR^36^ was used for relative quantification and statistical analysis of TMT-labeled peptides. All scans from unique peptides were considered for quantification.

Gene ontology (GO)^37^, KEGG orthology^38^ and BioGPS^39^ databases were used for biological categorization of the identified proteins that were significantly increased or decreased in AP conditions.

### Statistical analysis

Statistical analysis was performed with GraphPad Prism software. Data are presented as mean ± S.E.M. Data were analyzed using a two-tailed Student’s t-test for comparison of two groups, and one-way analysis of variance (ANOVA) analysis followed by Tukey’s post-test when comparing three groups. Statistical significance was considered when *p* < 0.05.

For mass spectrometry analysis, ion reporter intensity data was normalized using QuantileN normalization. DanteR ANOVA was performed at protein level by comparing treated versus control peptides using a linear model and the *p*-values were adjusted by using the Benjamini & Hochberg False Discovery Rate correction. Regulated peptides were determined using an adjusted *p*-value cutoff of 0.05 and a fold change lower than 0.67 (down) or higher than 1.5 (up).

## Supplementary information

Table S1

Table S2

Table S3

## References

[1] Peery AF (2019). Burden and Cost of Gastrointestinal, Liver, and Pancreatic Diseases in the United States: Update 2018. Gastroenterology.

[2] Banks PA (2013). Classification of acute pancreatitis–2012: revision of the Atlanta classification and definitions by international consensus. Gut.

[3] Singh VK, Moran RA, Afghani E, de-Madaria E (2015). Treating acute pancreatitis: what’s new?. Expert Rev. Gastroenterol. Hepatol..

[4] Hirota M (2000). Relationship between plasma cytokine concentration and multiple organ failure in patients with acute pancreatitis. Pancreas.

[5] Gutierrez PT, Folch-Puy E, Bulbena O, Closa D (2008). Oxidised lipids present in ascitic fluid interfere with the regulation of the macrophages during acute pancreatitis, promoting an exacerbation of the inflammatory response. Gut.

[6] Folch, E. *et al*. P-Selectin Expression and Kupffer Cell Activation in Rat Acute Pancreatitis. *Dig. Dis. Sci*., **45**, 1535–1544 (2000). 10.1023/a:1005552725243 11007102

[7] Freise J, Schmidt FW, Magerstedt P, Schmid K (1985). Gabexate mesilate and camostate: new inhibitors of phospholipase A2 and their influence on the alpha-amylase activity in serum of patients with acute pancreatitis. Clin. Biochem..

[8] De Toro J, Herschlik L, Waldner C, Mongini C (2015). Emerging Roles of Exosomes in Normal and Pathological Conditions: New Insights for Diagnosis and Therapeutic Applications. Front. Immunol..

[9] Bonjoch L, Casas V, Carrascal M, Closa D (2016). Involvement of exosomes in lung inflammation associated with experimental acute pancreatitis. J. Pathol..

[10] Zhao Y (2016). Pancreatic Acinar Cells Employ miRNAs as Mediators of Intercellular Communication to Participate in the Regulation of Pancreatitis-Associated Macrophage Activation. Mediators Inflamm..

[11] Wu Y, Deng W, Klinke DJ (2015). Exosomes: improved methods to characterize their morphology, RNA content, and surface protein biomarkers. Analyst.

[12] Essandoh K, Li Y, Huo J, Fan G-C (2016). MiRNA-Mediated Macrophage Polarization and its Potential Role in the Regulation of Inflammatory Response. SHOCK.

[13] Liu DZ (2016). Elevating microRNA-122 in blood improves outcomes after temporary middle cerebral artery occlusion in rats. J. Cereb. Blood Flow Metab..

[14] Hsu S-H (2012). Essential metabolic, anti-inflammatory, and anti-tumorigenic functions of miR-122 in liver. J. Clin. Invest..

[15] Bandiera S, Pfeffer S, Baumert TF, Zeisel M (2015). B. miR-122 – A key factor and therapeutic target in liver disease. J. Hepatol..

[16] Fu D (2017). MiRNA-21 has effects to protect kidney injury induced by sepsis. Biomed. Pharmacother..

[17] Loboda A, Sobczak M, Jozkowicz A, Dulak J (2016). TGF- *β* 1/Smads and miR-21 in Renal Fibrosis and Inflammation. Mediators Inflamm..

[18] Zhou W (2018). MicroRNA-21 down-regulates inflammation and inhibits periodontitis. Mol. Immunol..

[19] Bejerano T, Etzion S, Elyagon S, Etzion Y, Cohen S (2018). Nanoparticle Delivery of miRNA-21 Mimic to Cardiac Macrophages Improves Myocardial Remodeling after Myocardial Infarction. Nano Lett..

[20] Wan J (2019). Inhibition of miR-155 reduces impaired autophagy and improves prognosis in an experimental pancreatitis mouse model. Cell Death Dis..

[21] Wang Z, Lieberman PM (2016). The crosstalk of telomere dysfunction and inflammation through cell-free TERRA containing exosomes. RNA Biol..

[22] Wang Z (2015). Telomeric repeat-containing RNA (TERRA) constitutes a nucleoprotein component of extracellular inflammatory exosomes. Proc. Natl. Acad. Sci..

[23] Cai TQ (2001). Induction of 11beta-hydroxysteroid dehydrogenase type 1 but not -2 in human aortic smooth muscle cells by inflammatory stimuli. J. Steroid Biochem. Mol. Biol..

[24] Chapman K, Holmes M, Seckl J (2013). 11β-Hydroxysteroid Dehydrogenases: Intracellular Gate-Keepers of Tissue Glucocorticoid Action. Physiol. Rev..

[25] Gulfo J (2016). New Roles for Corticosteroid Binding Globulin and Opposite Expression Profiles in Lung and Liver. PLoS One.

[26] Sternby Hanna, Bolado Federico, Canaval-Zuleta Héctor J., Marra-López Carlos, Hernando-Alonso Ana I., del-Val-Antoñana Adolfo, García-Rayado Guillermo, Rivera-Irigoin Robin, Grau-García Francisco J., Oms Lluís, Millastre-Bocos Judith, Pascual-Moreno Isabel, Martínez-Ares David, Rodríguez-Oballe Juan A., López-Serrano Antonio, Ruiz-Rebollo María L., Viejo-Almanzor Alejandro, González-de-la-Higuera Belén, Orive-Calzada Aitor, Gómez-Anta Ignacio, Pamies-Guilabert José, Fernández-Gutiérrez-del-Álamo Fátima, Iranzo-González-Cruz Isabel, Pérez-Muñante Mónica E., Esteba María D., Pardillos-Tomé Ana, Zapater Pedro, de-Madaria Enrique (2019). Determinants of Severity in Acute Pancreatitis. Annals of Surgery.

[27] Aho HJ, Suonpää K, Ahola RA, Nevalainen TJ (1984). Experimental pancreatitis in the rat. Ductal factors in sodium taurocholate-induced acute pancreatitis. Exp. Pathol..

[28] Folch E, Closa D, Prats N, Gelpí E, Roselló-Catafau J (1998). Leukotriene generation and neutrophil infiltration after experimental acute pancreatitis. Inflammation.

[29] Trush MA, Egner PA, Kensler TW (1994). Myeloperoxidase as a biomarker of skin irritation and inflammation. Food Chem. Toxicol..

[30] Shen, L., Hillebrand, A., Wang, D. Q.-H. & Liu, M. Isolation and Primary Culture of Rat Hepatic Cells. *J. Vis. Exp*. 10.3791/3917 (2012). 10.3791/3917 PMC3471302 22781923

[31] Shelke, G. V., Lässer, C., Gho, Y. S. & Lötvall, J. Importance of exosome depletion protocols to eliminate functional and RNA-containing extracellular vesicles from fetal bovine serum. *Journal of Extracellular Vesicles*, **3**, (2014). 10.3402/jev.v3.24783 PMC4185091 25317276

[32] Théry, C., Amigorena, S., Raposo, G. & Clayton, A. Isolation and characterization of exosomes from cell culture supernatants and biological fluids. *Curr. Protoc. Cell Biol*. Chapter 3, Unit 3.22 (2006). 10.1002/0471143030.cb0322s30 18228490

[33] Sáenz-Cuesta M (2015). Methods for extracellular vesicles isolation in a hospital setting. Front. Immunol..

[34] Wiśniewski JR, Zougman A, Nagaraj N, Mann M (2009). Universal sample preparation method for proteome analysis. Nat. Methods.

[35] Nguyen TD (2015). The phosphoproteome of Human Jurkat T cell clones upon costimulation with 1 anti-CD3/anti-CD28 antibodies. J. Proteomics.

[36] Taverner T (2012). DanteR: an extensible R-based tool for quantitative analysis of -omics data. Bioinformatics.

[37] Gene Ontology Consortium: going forward. *Nucleic Acids Res*. **43**, D1049–56 (2014). 10.1093/nar/gku1179 PMC4383973 25428369

[38] Kanehisa M, Goto S (2000). KEGG: kyoto encyclopedia of genes and genomes. Nucleic Acids Res..

[39] Siragusa L, Cross S, Baroni M, Goracci L, Cruciani G (2015). BioGPS: navigating biological space to predict polypharmacology, off-targeting, and selectivity. Proteins.

