# Supplementary material for: Acute pancreatitis promotes the generation of two different exosome populations
Source: Sci Rep. 2019 Dec 27;9:19887. doi: 10.1038/s41598-019-56220-5 (PMC6934470; doi:10.1038/s41598-019-56220-5)
Supplement: Supplementary file 3 — Table S3 [file 41598_2019_56220_MOESM3_ESM.docx]

**Acute pancreatitis promotes the generation of two different exosome populations**

Jiménez-Alesanco A^1+^, Marcuello M^2+^, Pastor-Jiménez M^1^, López-Puerto L^1^, Bonjoch L^1^, Gironella M^2^, Carrascal M^3^, Abian J^3^, de-Madaria E^4^, Closa D^1^*

**Supplementary information**

**Table S3: Primers used for RT-qPCR**

**Table S3: Primers used for RT-qPCR**

| **Primer Name** |  |
| --- | --- |
| IL-1β F | 5′-AAAAATGCCTCGTGCTGTCT-3′ |
| IL-1β R | 5′-TCGTTGCTTGTCTCTCCTTG-3′ |
| CCL2 F | 5′-TAGCATCCACGTGCTGTCTC-3′ |
| CCL2 R | 5′-TGCTGCTGGTGATTCTCTTG-3′ |
| CXCL1 F | 5’-GCGGAGAGATGAGAGTCTGG-3’ |
| CXCL1 R | 5’-AGGCATTGTGCCCTACAAAC-3’ |
| IL-6 F | 5′- CCGGAGAGGAGACTTCACAG -3′ |
| IL-6 R | 5′- CAGAATTGCCATTGCACAAC -3′ |
| 11βHSD1 F | 5’-TGCTCAGGACCACATAGCTG-3’ |
| 11βHSD1 R | 5’-TTTTGCAGAGCGATTTGTTG-3’ |
| 11βHSD2 F | 5’-GCAGCAGCTCTCTAGGCAAG-3’ |
| 11βHSD2 R | 5’-TCAAGGTCAGCATCATCCAG-3’ |
| GAPDH F | 5′-CTGTGTCTTTCCGCTGTTTTC-3′ |
| GAPDH R | 5′-TGTGCTGTGCTTATGGTCTCA-3′ |
